# Coronal extrusion of the lateral meniscus does not increase after pullout repair of the posterior root of the lateral meniscus at short-term follow-up

**DOI:** 10.1007/s00402-023-04815-z

**Published:** 2023-02-27

**Authors:** Philipp Forkel, Jonas Noack, Maximilian Hinz, Andreas B. Imhoff, Klaus Wörtler, Matthias J. Feucht

**Affiliations:** 1 Department of Orthopaedic Sports Medicine, Klinikum rechts der Isar, Technical University of Munich, Ismaninger Straße 22, 81675 Munich, Germany; 2 Raphaelsklinik, Münster, Germany; 3 Department of Diagnostic and Interventional Radiology, Klinikum rechts der Isar, Technical University of Munich, Munich, Germany; 4 Diakonie-Klinikum Stuttgart, Stuttgart, Germany

**Keywords:** ACL, Meniscus tear, Meniscus repair, Meniscus root repair

## Abstract

**Purpose:**

Posterior lateral meniscus root (PLMR) tears are injuries that commonly occur together with anterior cruciate ligament (ACL) tears. The aim of this study was to evaluate the clinical and radiological outcome of PLMR repair accompanying ACL reconstruction. Specifically, PLMR healing rates, meniscal extrusion behavior and their influence on patient-reported outcome measures (PROMs) were analyzed. It was hypothesized that PLMR repair shows satisfactory healing rates and coronal meniscal extrusion does not increase significantly following PLMR repair.

**Methods:**

Patients that underwent PLMR repair between 2014 and 2019 were evaluated at least 12 months postoperatively. At follow-up, magnetic resonance imaging (MRI) was performed in order to evaluate the PLMR healing behavior (complete vs. partial vs. no healing) as well as the coronal and sagittal meniscal extrusion in comparison with the preoperative MRI. Additionally, patient-reported outcome measures (PROMs; Lysholm score, International Knee Documentation Committee subjective knee form [IKDC]) were compiled. Pre- and postoperative meniscal extrusion were tested for statistical significance using the paired *t* test. The Kruskal–Wallis test was used to compare extrusion values and PROMs in relation to different healing states. A correlation analysis was conducted using the Pearson correlation coefficient between differences in meniscal extrusion and PROMs.

**Results:**

Out of 25 patients, 18 patients (72.0%; 11 male and seven female) were available for final assessment at a mean follow-up of 40.8 ± SD 17.5 months. One revision PLMR repair was performed five months after the initial repair. In 14 cases (77.8%), healing of the lateral meniscus was observed (6 × complete, 8 × partial). Coronal extrusion of the lateral meniscus did not increase significantly following PLMR repair (2.0 ± 1.5 mm vs. 2.1 ± 1.3 mm*; p* = 0.645). Sagittal extrusion increased significantly (25.7 ± 2.4 mm vs. 27.0 ± 1.4 mm*; p* < 0.001). The healing status of the PLMR showed no significant association with meniscal extrusion or PROMs (*p* > 0.05). But a higher increase in coronal meniscal extrusion negatively affected PROMs (Lysholm score: *p* = 0.046, *r* = − 0.475; IKDC: *p* = 0.003, *r* = − 0.651).

**Conclusion:**

High healing rates of the PLMR and no significant increase in coronal extrusion may be expected following combined PLMR repair and ACL reconstruction. But a greater increase in postoperative coronal meniscal extrusion correlates with less favorable clinical results. A greater increase in sagittal extrusion was observed, but this did not influence the clinical outcome.

**Level of evidence:**

Retrospective Case Series; IV.

## Introduction

Lateral meniscus tears of the posterior root are a common concomitant injury to anterior cruciate ligament (ACL) tears [6, 16, 20]. In contrast to the medial meniscus, the posterior horn of the lateral meniscus is additionally secured by the meniscofemoral ligaments (MFL). Radial or oblique tear configurations close to or within the meniscus root are often seen in posterior root tears of the lateral meniscus [16, 22]. A complete tear of the posterior lateral meniscus root (PLMR) and the MFL is rare but can occur [16]. Biomechanical studies revealed that isolated PLMR tears do not have the same destructive quality in terms of pressure distribution and destabilization of the lateral compartment compared to medial meniscus root tears and their effect on the medial compartment [14, 17–19, 27]. Posterior root tears of the medial meniscus are related to a rapid degeneration of the medial compartment and induce a medial osteoarthritis in 95%, if left untreated [5]. The consequence of a PLMR tear left in situ at the time of an ACL reconstruction may instead be biomechanically less consequential, especially in cases with intact MFL as only slight joint-space narrowing occurred ten years following ACL reconstruction and a concomitant PLMR tear left in situ [26]. However, an increase in lateral meniscal extrusion may be recognized in cases of PLMR and ACL tears on preoperative magnetic resonance imaging (MRI) [25]. Therefore, the repair of traumatic PLMR tears is accepted and recommended from several authors and, subsequently, significant effort has been put into improving stabilization procedures [1, 7–10, 12–15, 24].

Many studies report on the clinical, radiologic and arthroscopic outcomes of posterior medial meniscus root repairs and their healing rates. Following posterior medial meniscus root repair, an improvement of clinical results and function may be expected despite unsatisfactory healing rates [11]. Additionally, a recent study found lesser progression of osteoarthritic changes and subsequent arthroplasty after posterior medial meniscus root repair compared to meniscectomy and nonoperative treatment [3].

Clinical reports investigating the healing behavior after PLMR tear repair in combination with ACL reconstruction are rare. One study assessed the healing status of medial or lateral root repairs via second-look arthroscopy at the time of ACL reconstruction in patients undergoing a two-stage revision ACL reconstruction surgery. The authors found encouraging healing rates (82% overall) when a transtibial pullout technique was performed during the first step of the two-step procedure [4]. A recent study by Krych et al. [23] compared pre- and postoperative meniscal extrusion between posterior lateral and medial meniscal root tear repairs. They found a significantly increased extrusion following posterior medial meniscal root tear repairs, but did not find a significant increase in meniscal extrusion following repair of the PLMR [23]. Additionally, no progression of cartilage degeneration or subchondral bone abnormalities were observed during their 6-month follow-up investigation [23].

The purpose of our study was to investigate the radiological and clinical short-term results of PLMR tear repair combined with a one-step ACL reconstruction. Our initial hypotheses were that a PLMR pullout repair would lead to a high rate of PLMR healing and coronal meniscal extrusion would not increase significantly following PLMR repair.

## Material and methods

Patients who underwent PLMR repair between 2014 and 2019 were included for retrospective review at a minimum follow-up of 12 months. Furthermore, patients were eligible for participation in this study if a preoperative MRI was available and if they were at least 18 years old at the time of follow-up. Patients with high-grade osteoarthritic changes (Kellgren Lawrence grade 3 or 4), degenerative lateral meniscus lesions and/or other concomitant lateral meniscus lesions were excluded.

The study was approved by the ethics committee of the Technical University of Munich (reference number: 442/19S-SR) and conducted according to the Declaration of Helsinki.

### Surgical technique

The arthroscopic treatment was performed by experienced orthopedic sports medicine surgeons. The typical treatment consisted of an ACL reconstruction using either a hamstring or a quadriceps tendon autograft. Additionally, a tibial pullout repair of the posterior horn of the lateral meniscus was performed. The root was grasped using the Knee Scorpion^™^ suture passer (Arthrex Inc., Naples, USA), and a FiberWire Nr. 0 (Arthrex Inc., Naples, USA) was brought through the meniscus tissue. The tibial plateau was debrided at the root insertion, and the meniscus root was reduced to the tibial plateau by pulling the sutures through the tibial tunnel. Depending on the intraoperative situation and the individual anatomy, the pullout suture was either performed through an independent tunnel or together with the ACL graft through the tunnel prepared for the ACL reconstruction. The pullout suture was fixated together with the ACL graft using an interference screw in the tibial tunnel, an extracortical backup-fixation, or both.

### Postoperative rehabilitation

After surgery, the affected leg was secured in a knee brace (M.4 s^®^ comfort, medi GmbH & Co. KG, Bayreuth, Germany) for six weeks with knee flexion limited to 60°. During this time, weight bearing was not allowed (sole contact only). After a follow-up examination 6 weeks postoperatively, patients were encouraged to steadily increase the weight put on the operated leg until full weight bearing was achieved. Physiotherapy started on the first postoperative day, and patients received physiotherapy treatments 2–3 times per week.

### Outcome parameters

The single follow-up examination consisted of a clinical and radiological examination. In the clinical examination, side-to-side difference in anteroposterior stability was assessed using the KT-1000 arthrometer (MEDmetric Corporation, San Diego, California, USA), and patient-reported outcome measures (Lysholm score and the International Knee Documentation Committee subjective knee form [IKDC]) were documented. Additionally, postoperative MRI was performed at the Department of Diagnostic and Interventional Radiology at the Technical University of Munich on a 3 Tesla whole-body magnetic resonance scanner (Verio, Siemens, Erlangen, Germany) with the use of a dedicated eight-channel knee coil. The following pulse sequences were acquired: sagittal, coronal and axial intermediate-weighted turbo spin echo sequences (BLADE) with spectral fat saturation (ETL 9, TR 3300–5280 ms, TE 43–47 ms, FOV 160 mm, SD 3 mm) and a sagittal T1-weighted turbo spin echo sequence with a driven equilibrium (DRIVE) pulse for native arthrographic contrast (ETL 3, TR 790 ms, TE 16 ms, FOV 160 mm, SD 3 mm).

**Fig. 1:**
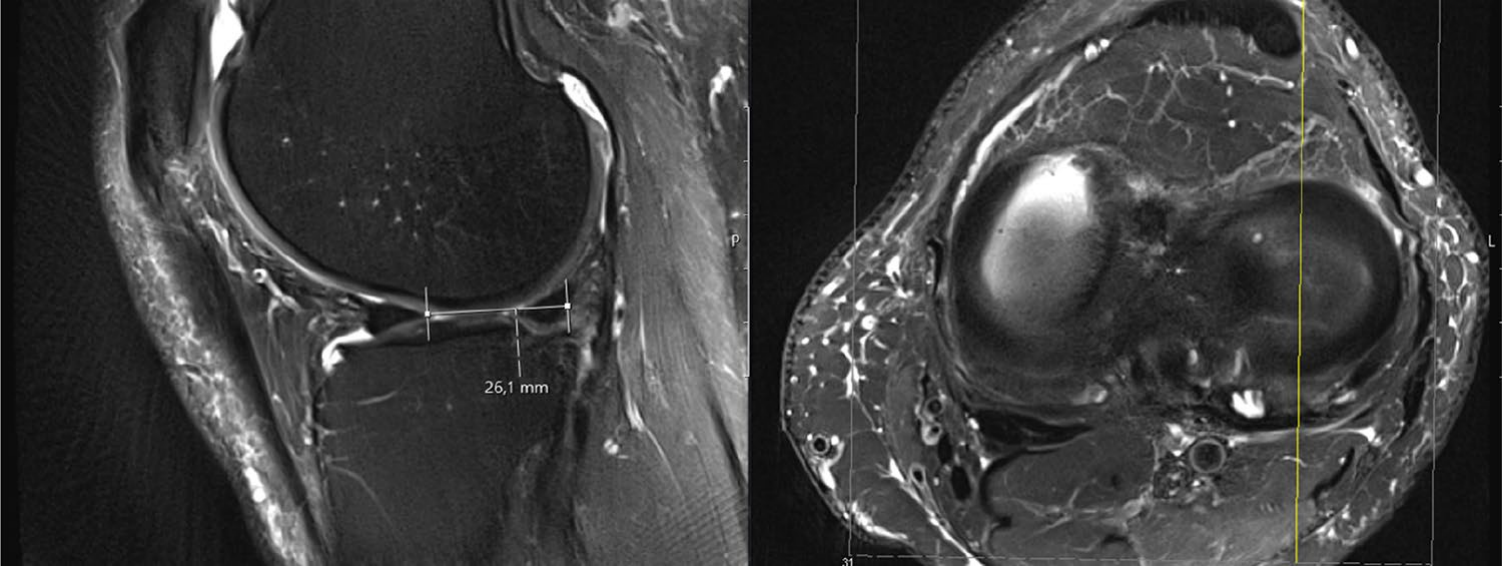
Magnetic resonance imaging. Measuring of the sagittal extrusion. The yellow line indicates the position of measuring. The distance between the inner margin of the lateral meniscus anterior horn and the posterior meniscocapsular junction was measured according to Ahn et al. [2]

**Fig. 2:**
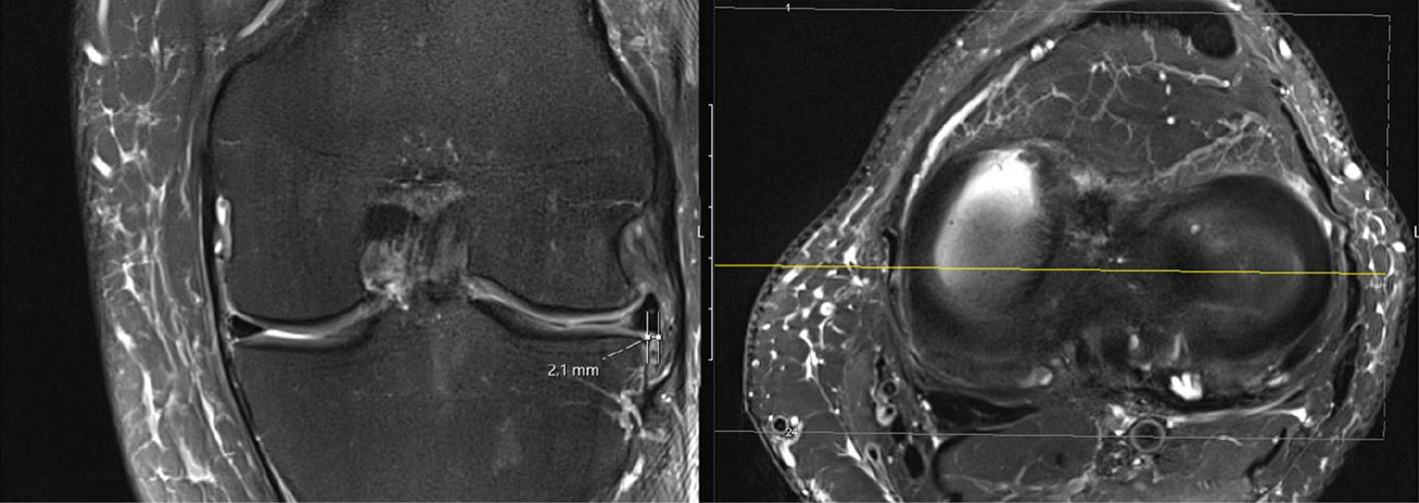
Magnetic resonance imaging. Measuring of the coronal extrusion. The yellow line on the axial plane indicates the position of measuring. According to Ahn et al. [2], the greatest distance of the meniscus from the peripheral margin of the lateral proximal tibial plateau to the meniscocapsular junction was measured on the coronal plane images

To guarantee pre- and postoperative comparability of the MRI scans and, subsequently, to receive reliable values of meniscal extrusion, the length of the patella was determined in the sagittal plane on both MRI. Then, a ratio was calculated to eradicate a possible measuring error due to different MRI modalities. The coronal and sagittal extrusion of the lateral meniscus were measured on preoperative and postoperative MRI. Every rater performed the measurements independently and selected the correct slices by himself. According to Ahn et al. [2], the greatest distance of the meniscus from the peripheral margin of the lateral proximal tibial plateau to the meniscocapsular junction was measured on the coronal plane images. The distance from the inner margin of the anterior horn of the lateral meniscus to the meniscocapsular junction of the posterior horn of the lateral meniscus was measured on the sagittal image (see Figs. 1 and 2). Postoperative MRI were evaluated in the sagittal, coronal and axial plane for lateral meniscus root healing. Healing was defined as “complete,” “partial” or “no healing” as previously described by Kim et al. [21]. Complete healing was defined as a complete contact between the meniscus and the osseous insertion, whereas partial healing was defined as contact between the meniscus and the osseous insertion site, but with a gap on at least one of the MRI planes. No healing was defined as no meniscus-to-bone contact detected on any of the planes. All pre- and postoperative MRI images were examined by two independent observers (KW and PF).

### Statistical analysis

Statistical analysis was performed using SPSS software version 25.0 (IBM-SPSS, New York, USA). Categorical variables are presented in sums and percentages. Normal distribution of the collected continuous variables was assessed by the Shapiro–Wilk test and graphically confirmed. Accordingly, continuous variables are presented either as mean and standard deviation or as median and 25–75% interquartile range. Pre- and postoperative meniscal extrusion were compared using the paired t test. The Kruskal–Wallis test was used to compare extrusion values, clinical scores and KT-1000 measurements in relation to different healing states. A correlation analysis was conducted using the Pearson correlation coefficient between differences in meniscal extrusion, anteroposterior stability and PROMs. To determine the interobserver reliability, intraclass correlation coefficients were calculated for all extrusion measurements. The level of significance was set at *p* < 0.05.

## Results

A total of 25 patients underwent a transtibial PLMR repair in the aforementioned time frame, of which 18 (72%) participated in the physical examination and underwent postoperative MRI. Due to large distances between the clinic and their place of residence, seven patients refused to take part in this study. The mean age of all included participants was 46.6 ± 11.2 years at follow-up, which was conducted 40.8 ± 17.5 months postoperatively. Eleven patients were male (61.1%) and seven patients were female (38.9%).

All patients underwent an arthroscopic PLMR repair at the Department of Orthopaedic Sports Medicine at the Technical University Munich. In 17 cases (94.4%), a simultaneous ACL reconstruction (15 × hamstring autograft, 2 × quadriceps autograft) was performed. In the remaining case (5.6%), a refixation of the tibial eminence was performed together with the PLMR repair. One patient (5.6%) who underwent ACL reconstruction with a hamstring tendon autograft together with the PLMR repair received revision surgery 5 months postoperatively; this was due to a dislocation of the suture material into the joint space and an associated audible grinding noise and pain. In this case, the root was re-ruptured and had to be fixated again with an individual tibial tunnel. No other complications occurred during follow-up.

The individual and different trauma patterns required a medial meniscus refixation in seven cases. In five cases, a fixation of the medial collateral ligament MCL was performed. One Larson reconstruction and one augmentation of the patellar tendon had to be performed.

### Magnetic resonance imaging

Complete healing of the PLMR was observed in six cases (33.3%). Eight cases (44.4%) showed partial healing, and four cases (22.2%) revealed a meniscus root insufficiency (no healing).

Coronal extrusion of the lateral meniscus did not increase significantly after PLMR repair (*p* = 0.645; see Table 1). Sagittal meniscal extrusion increased significantly following PLMR repair (*p* < 0.001; see Table 1).

**Table 1:** MRI extrusion measurements of the lateral meniscus. The sagittal extrusion increased significantly at follow-up, whereas the coronal extrusion did not increase significantly

	Sagittal MRI	Coronal MRI
Preoperative lateral meniscal extrusion	25.7 ± 2.4 mm	2.0 ± 1.5 mm
Postoperative lateral meniscal extrusion	27.0 ± 1.4 mm	2.1 ± 1.3 mm
Difference in lateral meniscal extrusion	1.3 ± 1.3 mm	0.1 ± 1.3 mm
*p* value	< 0.001	0.645

*MRI* magnetic resonance imaging

Normally distributed continuous variables are shown as mean ± standard deviation

Bolded *p* values indicate statistical significance

The correlation analysis between root integrity (complete healing, partial healing or no healing) and sagittal as well as coronal extrusion (pre- to postoperative extrusion difference and postoperative extrusion) did not reveal a significant correlation (*p* > 0.05; see Table 2 as well as Table 3). Patients with proven meniscus root integrity showed a reduced sagittal and coronal extrusion, but this trend was not statistically significant (*p* > 0.05). In summary, the pre- to postoperative extrusion difference, postoperative extrusion and postoperative PROMs were not significantly associated with the PLMR healing status evaluated by the postoperative MRI.

**Table 2:** Extrusion difference on sagittal and coronal MRI and their relation to the healing status of the meniscus root using the Kruskal–Wallis test

Meniscus root healing	Difference between pre- and postoperative sagittal extrusion (mm)	*p* value
Complete (*n* = 6)	0.9 ± 1.5	0.606
Partial (*n* = 8)	1.4 ± 1.5	
None (*n* = 4)	1.9 ± 0.9	

Meniscus root healing	Difference between pre- and postoperative coronal extrusion (mm)	*p* value
Complete (*n* = 6)	− 0.8 ± 1.0	0.145
Partial (*n* = 8)	0.6 ± 1.1	
None (*n* = 4)	0.5 ± 1.4	

*MRI* magnetic resonance imaging

Normally distributed continuous variables are shown as mean ± standard deviation

**Table 3:** Postoperative extrusion values on sagittal and coronal MRI and their relation to the healing status of the meniscus root using the Kruskal–Wallis test

Meniscal root healing	Postoperative sagittal extrusion (mm)	*p* value
Complete (*n* = 6)	27.3 ± 1.6	0.510
Partial (*n* = 8)	27.1 ± 1.5	
None (*n* = 4)	26.3 ± 1.9	

Meniscal root healing	Postoperative coronal extrusion (mm)	*p* value
Complete (*n* = 6)	1.7 ± 1.4	0.388
Partial (*n* = 8)	2.0 ± 1.5	
None (*n* = 4)	3.0 ± 1.6	

*MRI* magnetic resonance imaging

Normally distributed continuous variables are shown as mean ± standard deviation

The interrater reliability was “substantial” to “almost perfect” (κ range 0.766–0.926) for all extrusion measurements.

### Patient-reported outcome measures and anteroposterior stability

**Fig. 3:**
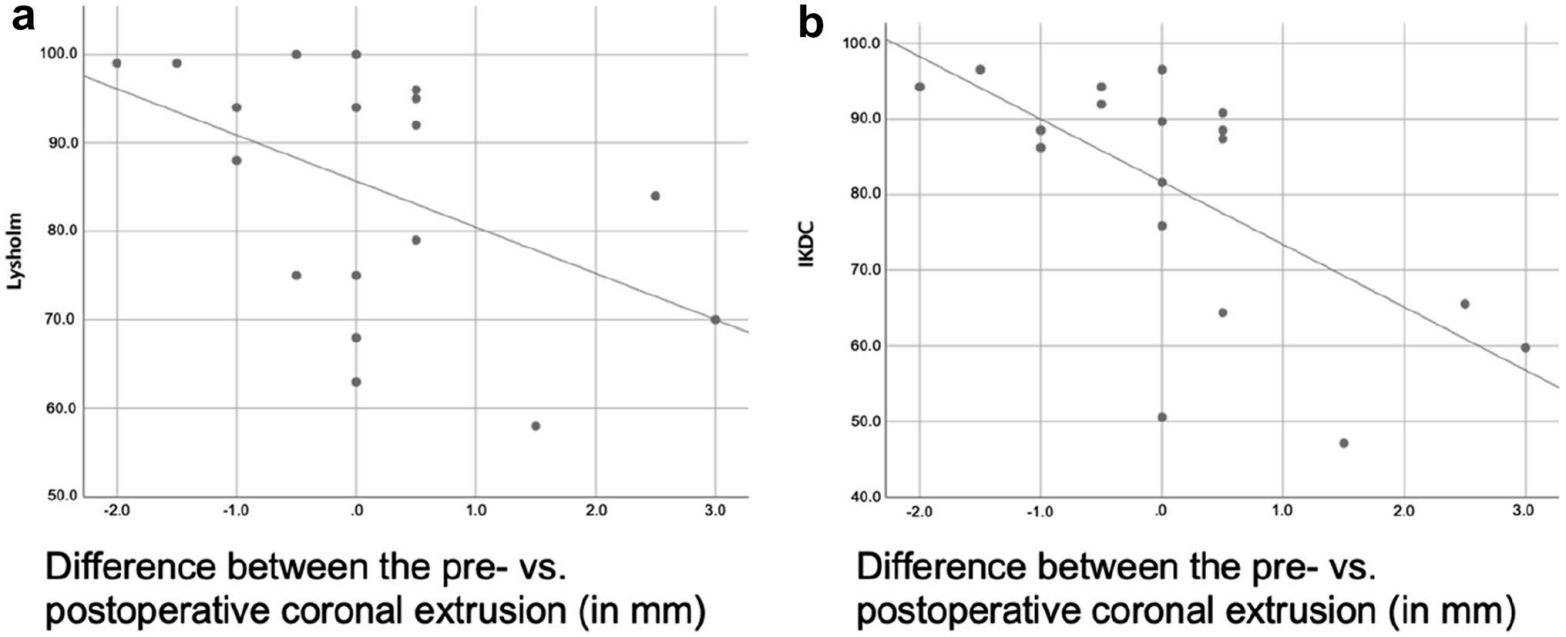
Pearson correlation coefficient between the differences in coronal extrusion (in mm) and the Lysholm score (**a**) and the IKDC (**b**). The diagram illustrates the development of a less favorable clinical outcome in the case of a greater increase in coronal extrusion (Lysholm score: *p *= 0.046, *r *= − 0.475; IKDC: *p *= 0.003, *r *= − 0.651)

Posterior lateral meniscus root healing status (complete healing, partial healing or no healing) was not associated significantly with the postoperative Lysholm score (*p* = 0.220), IKDC (*p* = 0.383) or anteroposterior stability (*p* = 0.288; see Table 4). The correlation analysis between the increase in pre- to postoperative sagittal and coronal meniscal extrusion and PROMs as well as anteroposterior stability revealed no significant correlation between meniscal extrusion and anteroposterior stability (*p* > 0.05). Also, a greater increase in sagittal meniscal extrusion did not affect PROMs (*p* > 0.05). A greater increase in coronal extrusion of the lateral meniscus, however, negatively affected PROMs (Lysholm score: *p* = 0.046 and *r* = − 0.475, IKDC: *p* = 0.003 and *r* = − 0.651; see Table 5 and Fig. 3).

**Table 4:** Patient-reported outcome measures and KT-100 arthrometer values and their relation to the healing status of the meniscal root using the Kruskal–Wallis test

Meniscal root healing	Lysholm score	IKDC	KT-1000
Complete (*n* = 6)	94.7 ± 4.3	90.2 ± 4.2	2.8 ± 1.6 mm
Partial (*n* = 8)	79.9 ± 15.0	77.0 ± 18.2	2.0 ± 1.7 mm
None (*n* = 4)	80.5 ± 15.6	73.0 ± 19.0	3.5 ± 1.3 mm
*p* value	0.220	0.383	0.288

*IKDC* International Knee Documentation Committee subjective knee form

Normally distributed continuous variables are shown as mean ± standard deviation

**Table 5:** Pearson correlation analysis between meniscal extrusion difference, PROMs and anteroposterior stability

Pearson correlation	Difference between pre- and postoperative sagittal extrusion	Difference between pre- and postoperative coronal extrusion
	*p* value	*p* value
KT-1000	0.641	0.564
Lysholm score	0.838	0.046
IKDC	0.450	0.003

*IKDC* International Knee Documentation Committee subjective knee form

Bolded *p* values indicate statistical significance

## Discussion

Our first hypothesis was proven with 77.8% (6 × complete healing, 8 × partial healing) of the included PLMR repairs showing complete or partial healing. The healing rate is similar to the rates reported by DePhillipo et al. (85%; [4]), but considerably lower than the healing rates observed by Krych et al. (98%; [23]) and Zhou et al. (100%; [28].

Following combined PLMR repair and ACL reconstruction (or ACL refixation), coronal meniscal extrusion did not increase significantly. But interestingly, these results were not significantly influenced by the healing status of the meniscal root.

Our findings complement those of a previous study by Krych et al. [23]. Zhou et al. [28], on the other hand, even reported a persistent reduction in the former coronal meniscal extrusion, which was not observed in this study.

Pre- to postoperative differences in coronal or sagittal meniscal extrusion were not affected by the healing status of the PLMR in our cohort. This is, however, in contrast to previous findings from Zhou et al. [28], who reported a greater reduction in meniscal extrusion as well as higher PROMs in patients with stable PLMR repairs, which was examined via second-look arthroscopy.

Regarding the healing status and its influence on PROMs, no significant influence was noted in our cohort, but the patients with complete healing did report the highest Lysholm score and IKDC values. Independent of PLMR healing, PROMs were satisfactory.

An increase in coronal meniscal extrusion, on the other hand, did negatively affect PROMs. The finding that the root integrity did not affect the meniscal extrusion in the coronal plane but the coronal extrusion negatively affects the clinical results might appear like a “paradox.”

To our interpretation, the coronal lateral extrusion indicates a loss of the lateral meniscus function in general. As previous biomechanical studies highlighted the impact of the loss function of the meniscofemoral ligaments [14, 17, 18], even damages to the popliteal fascicle and bruises to the meniscus body might contribute to a meniscal extrusion. On the other hand, the possible integrity of these structures might explain the absence of coronal extrusion even in the case of an absent root healing. Our findings underline the necessity to avoid an extrusion of the meniscus but do not indicate that a root repair solely helps to achieve this important goal. Our findings, instead, may highlight the importance of the different anatomic conditions of the lateral meniscal attachments and their role in stabilizing the lateral meniscus. In fact, the additional attachment of the lateral meniscus through the MFL and the shape of the lateral compartment may be the most important factors as previous biomechanical studies revealed the stabilizing factor of the MFL on the lateral meniscus and knee biomechanics.

The finding stresses the high importance of restoring the meniscus ring and avoiding meniscal extrusion in the coronal plane so that more favorable clinical outcomes may be achieved.

In our study, sagittal meniscal extrusion increased significantly, but this did not affect the clinical outcome. It may be concluded that the sagittal increase in the meniscal extrusion does not negatively affect the meniscus and its capability to contribute against axial load as well as maintain circular hoop tension. The potential positive effect of the MFL and the menisco-popliteal attachment is their contribution toward preventing meniscal extrusion. Even in cases of PLMR repair without healing evidence, they may limit meniscal extrusion, which is in line with the previous findings of Shelbourne et al. [26], who found only a small joint-space narrowing 10 years following PLMR tears left in situ.

However, findings in previous studies indicate that the preservation of the meniscus ring either with the intact meniscal root or the integrity of the MFL might be a relevant factor in order to avoid extrusion. Biomechanical studies describe a reduced negative influence on knee pressure distribution and knee rotational laxity [14, 17]. They indicated an increase in pressure and an increase in the rotational instability only in the case of a complete detachment of both the PLMR and the MFL. The repair of the PLMR seems to be reasonable but its influence on the restoration of the meniscus ring and avoidance of meniscal extrusion should not be overestimated.

### Limitations

The most important limitation of this study is the small number of included patients. Therefore, the findings of our study should not be overestimated and should be interpreted cautiously. In addition, the different and individual injury patterns and their necessary repair might influence the clinical results as reported with the PROMs specifically when considering the correlation analyses. The rate of complete or partial healing of the PLMR did not reveal a statistical significant relation with the postoperative meniscal extrusion; however, a bigger cohort might reveal such a relation. The anatomic condition of the lateral meniscus with its additional fixation via the MFL might contribute to the positive postoperative extrusion behavior, but this interpretation could not be verified with our analysis. Therefore, future studies should assess the intraoperative integrity of the MFL more thoroughly to prove or negate our interpretation.

## Conclusion

High healing rates of the PLMR and no significant increase in coronal extrusion may be expected following combined PLMR repair and ACL reconstruction. A greater increase in postoperative coronal meniscal extrusion correlates with less favorable clinical results. An increase in sagittal extrusion was observable, but this did not influence the clinical outcome.

## Data Availability

Data are with the corresponding author.
